# 25. OLIGODENDROCYTE-BASED IMPAIRMENT OF BRAIN CONNECTIVITY AS TARGET FOR NEW TREATMENT STRATEGIES IN SCHIZOPHRENIA

**DOI:** 10.1093/schbul/sby014.100

**Published:** 2018-04-01

**Authors:** Johann Steiner

**Affiliations:** University of Magdeburg

## Abstract

**Overall Abstract:** This symposium has a translational approach. First, we present human post-mortem and in-vivo imaging studies on the pivotal role of oligedondrocyte loss and dysfunction with consecutive impairments of brain connectivity in schizophrenia. Natalya Uranova will show morphometric data on ultrastructural alterations of oligodendrocytes, myelin damage and degeneration and disturbed oligodendrocyte-axon interactions in post-mortem prefrontal white matter in schizophrenia. Adrienne Lahti will report diffusion tensor imaging data suggesting impaired axonal and myelin integrity. Because, MR Spectroscopy permits the non-invasive measurement of neurometabolites, such as N-acetylaspartate, a marker of neuronal integrity, and glutamate, which can be neurotoxic when overproduced, this technique provides further understanding of the relationship between white matter microstructure and neuronal function.

Second, we present data from cell culture and animal models suggesting that restoration of oligodendrocyte function (in terms of energy metabolism, maturation and myelin production) is a promising target for the development of novel treatment strategies in schizophrenia. Proteomic studies in postmortem brain by Daniel Martins-de-Souza have suggested a schizophrenia-related energy metabolism dysfunction in oligodendrocytes. These findings have been followed up using oligodendroglia cell lines and induced pluripotent stem cell-derived cerebral organoids, supporting the notion that alterations in glycolysis in oligodendrocytes are pivotal to the overall energy dysfunction in schizophrenia brains. Lan Xiao′s lab has shown that oligodendrocyte dysfunction and impaired myelination in the prefrontal cortex is correlated with schizophrenia-like behavior in mice undergoing prolonged social isolation. Enhancing oligodendrocyte generation and myelin repair by FDA-approved compounds, like quetiapine (an APD) or clemastine (a histamine antagonist) successfully reversed the above phenotype.

